# Potential Gene Interactions in the Cell Cycles of Gametes, Zygotes, Embryonic Stem Cells and the Development of Cancer

**DOI:** 10.3389/fonc.2015.00200

**Published:** 2015-09-23

**Authors:** Gregor Prindull

**Affiliations:** ^1^Medical Faculty, University of Göttingen, Göttingen, Germany

**Keywords:** gene transcription factors, gametes, zygotes, embryonic stem cells, pre-neoplastic

## Abstract

**Objectives:**

This review is to explore whether potential gene interactions in the cell cycles of gametes, zygotes, and embryonic stem (ES) cells are associated with the development of cancer.

**Methods:**

MEDPILOT at the Central Library of the University of Cologne, Germany (Zentralbibliothek Köln) that covers 5,800 international medical journals and 4,300 E-journals was used to collect data. The initial searches were done in December 2012 and additional searches in October 2013–May 2015. The search terms included “cancer development,” “gene interaction,” and “ES cells,” and the time period was between 1998 and 2015. A total of 147 articles in English language only were included in this review.

**Results:**

Transgenerational gene translation is implemented in the zygote through interactions of epigenetic isoforms of transcription factors (TFs) from parental gametes, predominantly during the first two zygote cleavages. Pluripotent transcription factors may provide interacting links with mutated genes during zygote-to-ES cell switches. Translation of post-transcriptional carcinogenic genes is implemented by abnormally spliced, tumor-specific isoforms of gene-encoded mRNA/non-coding RNA variants of TFs employing *de novo* gene synthesis and neofunctionalization. Post-translationally, mutated genes are preserved in pre-neoplastic ES cell subpopulations that can give rise to overt cancer stem cells. Thus, TFs operate as cell/disease-specific epigenetic messengers triggering clinical expression of neoplasms.

**Conclusion:**

Potential gene interactions in the cell cycle of gametes, zygotes, and ES cells may play some roles in the development of cancer.

## Introduction

DNA-encoded gene replication is usually stable but not permanent during meiotic and mitotic cell cycles. Indeed, the genome can be interpreted as an evolutionary organ of epigenetic gene transcription/translation; its complex gene replication patterns affect normal, as well as neoplastic cells (1, 2). The circumstances that lead to gene plasticity of normal cells, and neoplastic translational expression of mutated genes still are in part enigmatic. They may be initiated during development of embryonic stem (ES) cells that are in bi-directional dynamic equilibria with their primordial germline stem cells (PGCs). ES cells receive both normal and neoplastic transgenerational information from their parents via gametes that interact with each other in the zygote. Genes of gametes, zygotes, ES cells, and cancer stem cells (CSCs) interact through chromatin histone-mediated transcription factors (TFs) with or without involving environmental risk factors, such as biotic stress. Pluripotent TFs (ppTFs) are essential for unlimited proliferation and self-replication as epigenetic modulators in normal embryonic and neoplastic stem cells.

Mutagenic and carcinogenic properties of somatic cells are acquired by gene neosynthesis and neofunctionalization through alternative splicing of enzymatic messenger-/non-coding RNAs (m-/ncRNA) to isoforms that produce neoplastic effector proteins for translation (3, 4). It is not clear whether reactivation of silenced ES cells is involved in these processes (5, 6). Because ppTFs-mediated epithelial–mesenchymal/mesenchymal–epithelial transitions (EMT/MET) play major roles in both ES cell development and CSC transformation (7, 8), it is conceivable that EMT is an embryonic link to neoplastic transformation. This review hypothesizes that the role of TFs in genetic interactions of gametic, zygotic, and ES cells may be related to neoplastic transformation in somatic cells.

## Gene Translation in Cancer Etiology

Gene translation is the final step in phenotypic expression of post-transcriptional genes and is therefore important in cancer formation and prevention. It is implemented in loops of interacting networks of epigenetic TFs involving alternatively spliced mRNA/ncRNA isoforms and effector proteins through binding sites at cis-reacting imprinting control regions (ICRs) and UTR (untranslated gene regions). Research on potential interactions between/among genes involved in gene translation in normal and neoplastic cell replications may help elucidate the mechanisms of cancer etiology and treatment.

### Epigenetics starts in parental gametes

Epigenetic TFs control mitotic cell cycles as drivers. They originate in parental gametes and are transmitted transgenerationally to the fertilized ovum. In the zygote, pronuclear TFs interact with, and translate, genes of their fusion partners, trigger cleavages of the zygote, cause zygote-to-ES cell switches, and induce proliferation/differentiation of diploid gamete-to-zygote-to-embryonic stem (GZES) cells. It is now well established that parental occupational exposure to environmental carcinogens (9) causes damage to proliferating embryonic/germline cells as mutations and enzymatic mitotic lesions. Toxicants include industrial chemicals, such as dioxine and polychlorite biphenyles pesticides, and pharmaceutical products, e.g., cytostatic drugs that block gene transcription and may cause permanent damage in blastocysts. Even pre- and peri-conceptional exposures are associated with disease in the offspring through interfering with meiotic and/or mitotic cell cycles (10, 11). For example, alterations of temporal and spatial patterns of meiotic DNA replication are induced through involvement of proteins SMC1β, RAD21L, and STAG3 of the TET1-regulated replication machinery by complex cohesion links between meiosis and ­cancer (12). Lesions affecting embryonic CpG methylation patterns modify histone structures of endocrine disruptors, generate ­pre-neoplastic ES cells, and contribute to post-natal neoplastic cell growth (13). It is not known whether zygotes have the ability to “learn” and adopt biochemically to genotoxic stress. Importantly, toxic environmental effects may be inheritable across consecutive generations even without direct continuing environmental exposures (14, 15).

### Memory

Memory is based on transcriptional decisions of parental genes on events of the past that are mitotically transmitted through TFs to ES cells. This requires erasure of preceding/ancestral DNA-CpG transcriptional patterns through excision-glycosylases converting DNA-CpG-5mC (5methyl-cytosine) to 5hmC (5hydroxy-methyl-cytosine), to 5cC (5carboxyl-cytosine) and, finally, to thymine (16, 17). Modulating TFs enzymes are ten eleven translocation proteins (TET), autoinflammatory disease proteins (AID), methyl-CpG binding domain4 (MBD4), and DNA damage-inducible 45 protein (Gadd45). TETs1–3 are involved in converting CpG 5mC to 5hmC. AID operates via base excision repair pathways. MBD4 is an intermediate receptor; and Gadd45 is a response demethylase in DNA damage (18). Following erasure, parental memory is re-introduced to the ES cell genome by genomic programing. Incomplete or faulty transmission of memory information can result in abnormal gene transcription and post-natal diseases, including transgenerationally inheredited carcinogenic predispositions.

### Maternal effect genes

Transcriptional incompatibilities between parental pronuclei are silenced by histone-modulations during interactions in the zygote ensuring transcription stability in ES cell development. During the first two zygotic cleavages, reprograming is under signaling dominance of maternal gonad-specific factor (GSF)–maternal effect genes (*MEGs*) (19). The first two pre-ES cell cleavages of the zygote are executed by *MEG*-dominated TFs. *MEGs* are epigenetic genes transcribed and translated during oogenesis, and transmitted to the zygote by the maternal gamete. In fact, preformed *MEGs* contribute largely to the earliest biochemical steps of zygote development, in particular during passage through the oviduct. In the human embryo, both maternal and paternal zygote gene activation (ZGA) at the 4–8 cell stage is implemented through *MEG*-dominated alterations of the CpG histone methylation status by gene-encoded methyltransferase/demethylases (*DNMT/DM*) (20, 21). *MEGs* operate in zygotic reprograming through numerous maternal factors including cis-acting ZAR1, 2 (zygote arrest)/ZAR-like (ZARL) proteins in translational control sequences (TCS) that bind to maternal mRNAs at 3′UTR (22). Mutant ZAR1 arrests late 2-cell-stage zygotes through abnormal methylation of histones H3K4/H3K9 (histone H3 lysine4/9) and downregulate chromatin-modifying genes *Dppa* (maternal factor Stella, peri-plasmic polypeptide haloalkane dehydrogenase) and Piwil2 (protein of the ARGONAUTE family) (23). Also involved are Akt-PI3K (phospho-inositide-3-kinases) and genes *Mil1/Blimp1* (transcription repressors of somatic genes *Hox* and *Snail*). Maternal modulating TFs include cis-reacting RNA-binding proteins (RBPs), cytoplasmic polyadenylation elements (CPEs), and “deleted-azoospermia-like” (DAZL) family. CPEs mediate adaptation of gonadal homeostasis, metabolism, gene transcription of cell cycle cyclins, and apoptosis. DAZL is an mRNA translational component that controls RBP networks in the initial stages of zygote development until the embryonic genome is composed at the third post-fusion cleavage (24, 25). In addition to zygote reprograming, *MEGs* affect CTCF-DNA binding sites for paternal–maternal gene interactions at ICRs insulator sites (26) and licencing processes (21). CTCF is a zinc finger CCCTC repressor protein for gene regulation. Other *MEG* targets include somatic and imprinted genes, such as *Nlrp1/2* (early zygote, blastomere apoptosis), *Nlrp5* (Mater), subcortical maternal complexes (*SCMC*), *Nlrp14* (nucleotide-binding oligomerization in early embryonic development), Hsf1 (heat shock), *Npm2* (nucleoplasmin), *Cdh1* (e-cadherin), *Pms2* (mismatch repair gene2), *Ezh2* (enhancer of zeste, essential for ES cell self-renewal), and Smarca 4 (*Brg1*).

Clearly, *MEGs* exert fundamental influences on the development of the ES cell genome. They transmit genetic/epigenetic maternal memory information to the early stages of zygote-to-ES cell switches (27). They establish specific transgenerational transcription links for embryonal genes between parent memory and imprinted genes from PGCs (26, 28, 29). *MEG* mutations and functional distortions cause embryonic arrest at different developmental stages. Mutant genes *Npm2, Dppa3, Zar1*, and *Hsf1* arrest one-cell zygotes, while mutant *Dppa3, Pms2, Dnmt3a*, and *Dnmt1o* arrest later stages. *MEG* failures can cause carcinogenesis labeled by exposure-specific biomarkers for transgenerational disease and parental environmental exposures (9).

### Binding sites

Binding sites are gene-specific molecular moieties through which genetic/epigenetic partners interact with one another (6). Binding capacities are inherent properties of TFs for epigenetic control of gene transcription/translation. They operate through mRNA, ncRNA, and ribosomal proteins in regular as well as in pre-neoplastic mitotic cell cycles and are particularly important in clinical expression of neoplasms. TFs binding properties are not fixed. Rather, they are variable in embryonic, post-natal, and evolutionary development (30). Binding patterns vary in strength, are thermodynamically sensitive, and adapt to intra-/extracellular epigenetic stimuli. Novel genes have their own binding profiles. Binding properties to proteins are important in drug-design studies for molecular docking in structural identification of functional sites. Targeted inhibition of binding sites could serve therapeutic and preventive purposes for specific diseases, including cancer (31, 32).

### Untranslated gene regions (UTRs)

UTRs are a distinct, structurized class of non-coding, mostly cis-reacting RNA sequences that synergize with gene-specific mRNA-binding sites for a wide range of protein effectors (33). 3′/5′-UTR proteins and small ncRNAs control the flux of translation relevant information from the transcriptome to proteomes. They promote mRNA stability associated with protein-coding sequences at terminal endings of mRNA and of DNA-modifying histone genes (34), and regulate equilibria of interacting TFs with suppressors *p53* and cyclin D1 (*CCND1*) (35, 36). Interaction specificities of mRNA-alternative polyadenylation (APA)/isoform splicing are regulated by five histone H2A genes at UTR sites. 3′UTR, in particular, exerts multiple functions in carcinogenesis. It controls isoform splicing, activates signaling transduction of mutated gene cascades, determines mRNA-bindings to proteins, affects protein coding, and thus determines the type, direction, and translation of neoplastic transformation.

### Cis-reacting imprinting control regions

Genes interact with enzymatic TFs at ICRs. ICRs are transcriptional/translational sites composed of repetitive, germline-derived, differentially methylated DNA sequences on chromosomes positioned between eu- and heterochromatin. They function as insulators in genomic reprograming by delimiting acetylation-mediated barriers against allele-specific interactions of somatic as well as imprinted genes (26). ICRs separate active TFs in euchromatin from neighboring heterochromatic, silencing regions. Silencing occurs in synergisms with silent-information regulator (SIR)-mediated disruption of transcription activator complexes and RNA polymerase (Pol) II that blocks translation through elongation barriers on nucleosomes (37). Transcriptional chromatin lesions are partially compensated at ICRs through repression of histone H3K4me3 (lysine4 trimethylated histone H3) and polycomb complex1-mediated mixed lineage leukemia2 gene (MLL2). Distorted insulators are pathogenetic in post-natal diseases, including leukemogenesis, most likely through leakages between hetero- and euchromatin domains of chromatin modulators (38).

## Transcription / Translation Factors

Genes transcribed in mitoses become functional and exercise their properties through translation to effector proteins.A multifunctional mRNA-/polymerase A-binding protein (PABP1) serves as scaffold for protein–protein interactions. Production of effector proteins is mediated/controlled by translational TFs-mRNA/ncRNA. Indeed, unless genes are translated, genes are silenced and stored in heterochromatin. Thus, gene transcription and translation are interdependent but distinct and consecutive processes that can be separated by the specificities of their enzymes. Translating TFs include lysyl-tRNA synthetase (lysRS), isoform1 of translation elongation factor eEF that adjusts transcriptional yields to translational needs by associating with elongating RNA Pol II at 3′UTR, and cell fate effector DACHS. The latter mediates cytoplasmic oncogenic translation (for example in EMT) by affecting Y box-binding snail proteins. Clearly, it is translation that directs phenotypic expression of transcribed DNA sequences and therefore decides on the phenotypic, clinical expression of carcinogenic genes.

Pre-mRNAs are formed from DNA templates at UTRs. They are specified by APA and RNA-recognition motifs (RRMs) for splicing into isoforms [see below, Ref. (39)] with characteristic RBPs (40). Specificities are gene encoded through exon-binding (5). Different isoforms from a single mRNA control specified phases of diploid zygote-to-GZES cell switches (ZGS) in normal and carcinogenic mitoses, thus directing gene development into distinct directions (29). After transport to the cytoplasm, mature RNA isoforms are stored in cytoplasmic granules (41, 42). Enhancer export factor Np13 (a serine–arginine-rich shuttling protein) and DEAD-box RNA nucleo-cytoplasmic Dbp5 helicases release mRNA isoforms from storage at distinct times for DNA translation. Dbp5s are export factors for ATP-dependent remodeling. Translation is terminated by endo-ribonuclease RNA-E-dependent ncRNAs (40, 43, 44). Importantly, mRNAs are indispensible and frequently overexpressed in carcinogenesis and EMT-mediated metastatic dissemination (7, 8, 11, 12, 26, 36).

An important group among TFs is non-coding RNAs (ncRNA). They enzymatically regulate pre-mRNA splicing and edit mRNA-mediated gene transcription/translation (43, 45). Editing consists in interactions with poly (A) binding proteins PAN2/3, adenosine-to-inosine (A-to-I) converting enzymes, and CCR4-NOT de-amylase complexes (46, 47). Two major groups of ncRNAs are distinguished at 3′ and 5′ ribosome-binding sites (RBS). Group I uses guanosine, and group II employs the 2-OH group of internal adenosine. Both affect stability, biogenesis, and target recognition of mRNAs and can be pathogenetic in human disease. ncRNA regulators include DNA methyltransferases (DNMT), histone deacetylases, and polycomb group genes (6). They bind to more than one molecular mRNA domain forming enzyme complexes that compete in crosstalks with several different mRNAs (48, 49). Abnormally expressed ncRNA often are associated with a poor clinical outcome in cancer patients. Because ncRNA usually target entire translation pathways, they may be more effective therapeutic targets for enzyme inhibitors than mRNA genes or proteins.

ncRNAs comprise several functionally defined subgroups, including small (20–30 nucleotide)/small interfering RNAs (s/siRNAs) (43, 50, 51), long (more than 200 nucleotides) RNAs (lncRNAs), piwi transposion/retrovirus interacting RNA (piRNA) (5), and others (52). The subgroups have different functions: sRNAs edit high-grade myelodysplastic syndromes (MDS) via transfer RNAs (tRNAS) and may provide potential links to apoptosis (53). lncRNAs modify translating mRNA affecting proliferation, invasive motility, and survival of transcribed genes. They are frequently involved in carcinogenesis competing with endogenous enhancer-like RNAs (ce/eRNA); guide histone lysine methyltransferases through H3K27me3 in enhancer of zeste homolog *(EZH1)* pathways with functional overlaps in EMT-coding pathways; and, importantly, are involved in stem cell pluripotency (54). Secondary protein bindings of lncRNAs differ in RNA-protein vs. DNA-protein interactions and in modulating gene expression programs. For example, lncRNA HOTAIRM1 (encoded in the human *HOXA* gene cluster) is a highly specific regulator for gene expression in switches from granulocytic proliferation to maturation phases in integrin-controlled cell cycles. Furthermore, lncRNAs control gene transcription by recruitment of silencing complexes to homology-containing loci of the genome. Thus, lncRNAs are important in embryonic development and in the pathogenesis of neoplastic diseases (55).

**Figure 1:**
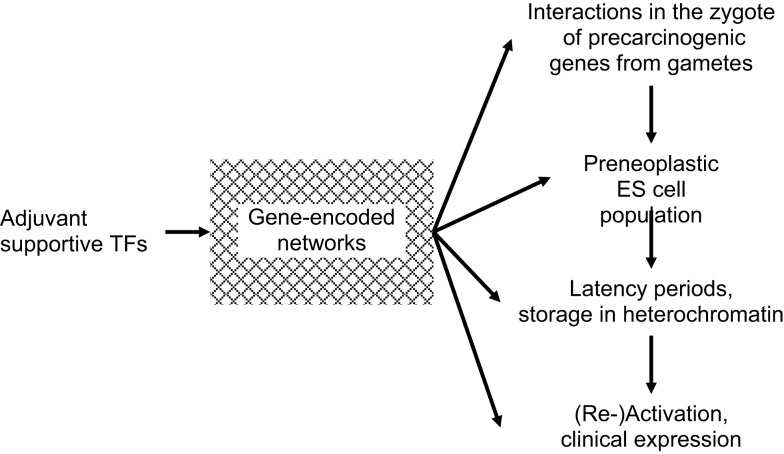
**Requirement of “adjuvant” transcription factors (TFs) at all stages of carcinogenesis**. Until/unless adjuvant networks are complete and ready for support of synergetic carcinogeneic (re)activation of cancer stem cells (CSCs), mutated genes will not be phenotypically expressed.

An additional group of indispensible TFs, characterized as being “adjuvant,” is shown in Figure 1. Adjuvant TFs are encoded predominantly by metabolic genes. They meet the regional requirements for survival and development of normal and pre-neoplastic cells in particular during transformation into fully neoplastic cells. Adaptations include supplies in nutrition, oxygen, and energy, for motility/angiogenesis, etc. In pre-neoplastic ES cells, adaptive metabolic alterations may be initiated already in the zygote before the first ES cell mitosis and continue into embryonic and post-natal development. Adjuvant support must be specific, quantitatively sufficient, and well synchronized with the appropriate mitotic stages. This applies in particular to phenotypic expression of clinical cancer, such as acute leukemias, and to EMT/MET in metastatic dissemination of solid tumors. Thus, adjuvant TFs are involved in the entire course of normal and neoplastic mitoses. Unless and until supportive, adjuvant TFs networks are complete and ready for synergetic activation of CSCs, mutated genes will not be phenotypically expressed. Rather they will be preserved in a pre-clinical state stored in heterochromatin from which they may be erased or await conversion to overt disease at some later time. Adjuvant adjustments may contribute to latency periods between infliction of genomic injury and neoplastic transformation and frequently extend over periods of years. Therapeutic blockage of adjuvant supportive networks might cause transcribed ­carcinogenic genes to remain untranslated.

### Alternative splicing to isoforms

TFs are enzymes that do not catalyze their targets directly. Rather, they control protein production indirectly through isoform copies that are produced by alternate splicing. In fact, 90% of gene-encoded mRNAs and ncRNAs undergo alternative splicing of their exons (the information-bearing parts of the gene) (56). APA is an integrated part of the splicing machinery that selectively protects poly (A)-tails of pre-mRNA from enzymatic digestion. It is bound to chromosomes, mostly within ±20 base pairs up- and downstream of splicing sites. Fip1, a gene-encoded subunit of the polyadenylation specificity factor CPSFs, is a key regulator of proximal polyadenylation at 3′-UTR-mRNA endings. It faithfully maintains distances to neighboring DNA sequences through insulators and promotes the production of shortened mRNA isoforms for pluripotency/renewal in GZES cells and in neoplastic transforming cells. Copies of mRNAs/ncRNAs are predominantly spliced by histone H3K36 methylation. Specific cytoplasmic cleavage factors include polyadenylation binding proteins (CPEBs) and catalyst-stimulating histone factors (CPCFs) (57). Specification of isoform targets is achieved by Pols II/III-splicing at C-terminal sequences (58, 59). RNA Pol II acts as an RNA-dependent RNA Pol. It initiates gene-specific protein synthesis and extends/destabilizes ncRNAs (60). Pol III regulates neofunctionalization of ancestral genes (61). Frequently, pre-mRNA is spliced into more than one isoform. For example, Nanog mRNA, a homeodomain TF for ES cell pluripotency and self-renewal (see below), is expressed through several isoforms arranged at 3′-UTRs in tandem nucleotides of four alternative transcription- and five different polyadenylation-start sites. Occasionally, pre-mRNA transcripts are translated into isoforms with opposing functions. An example is vascular endothelial growth factor (VEGF) isoforms that have pro- as well as anti-angiogenic properties (62).

Alternatively spliced TFs promote the flow of genomic information between transcription and translation. mRNA/ncRNA isoforms coordinate transgenerational gene activation via zygote-to-ES cell switches and in GZES cell physiology (28, 63, 64). Differently spliced TFs isoforms alter functional diversity and stability in distinct ways that extend TFs properties. Alterations in close proximity to exon/intron boundaries may weaken recognition sites of natural splice acceptors or donors and lead to exon skipping isoforms at cryptic splice sites. Importantly, TFs isoform splicing produces gene duplications with epigenetic properties deviating from the original. Leeway copies greatly enlarge the functional scope of TFs. They can induce gene neosynthesis (discussed below) and neofunctionalization of pre-existing, silenced (ancestral) DNA sequences endowing them with new CpG methylation patterns. Neofunctionalization employs nucleolin and Dbp2. Nucleolin acts with RNA regulating motif boxes RRM/RGG for transcriptional repeats, while Dbp2s (RNA-dependent helicases) are co-transcriptional modulators for clearance of genomic loci from preceding translations. Dbps control the quality of mRNA structures and ribonucleoprotein complexes, allow the formation of new chromatin and mRNPs (ribonucleoprotein) assemblies (65), repress aberrant DNA transcription, and promote transcription fidelity. Loss of Dbp2 leads to failure of chromatin structuration and distortion of gene expression. Neofunctionalization may occur already during embryonic development (66), e.g., by elimination of dinucleotides GT or AG at the 5′ and 3′ ends of introns (13, 41, 67). Isoforms may have abnormal exon lengths and a leaky structuration (38, 56). Indeed, distorted C-terminal sequences are caused in promyelocytic leukemias by abnormally spliced isoforms that silence tumor-suppressor genes *p53/p63/73* (68, 69). Other variants cause genomic instability and chromsome aneuploidy, initiate abnormal protein synthesis and may thus act as carcinogenic factors (70).

### Isoform splicing in clinical carcinogenesis

Splicing of TFs to isoforms plays a central role in clinical oncogenesis because it can induce gene neogenesis and neofunctionalization of silenced ancestral genes. The former is discussed in the section below. Alternatively spliced carcinogenic isoforms specifically direct the translation of mutated genes, but the mechanisms of selection for splicing to carcinogenic isoforms are still enigmatic. Numerous clinical carcinogenic pathways, such as *MEK, RAS, and ERK/RAF*, employ carcinogenic isoforms of high-order chromatin structures that promote neoplastic transformation. Also involved in deregulated translation by splicing are mRNA kinases such as RUNX1, CBFβ, MLL, C/EBPα, SPI1, GATA, and TAL1 that may cause misplacement of transcription factor binding-/start sites (TFBS/TSS) (71, 72). For example, isoform elF4H1 (spliced from translation-initiation elF-mRNAs) activates oncogenic signaling for cell proliferation (73). Other isoforms activate dormant mRNAs by opening their elongated carcinogenic poly (A) tails. Pre-mRNA variant isoform C5-V6-C6 of alternatively spliced exons of CD44 (67) (a cell membrane glycoprotein mediating cellular responses to the microenvironment) confers metastatic potentials to CSCs in EMT (74). This is interesting because EMT–TF complexes are involved in the development of both normal and carcinogenic ES cells. It suggests that patients with identical cytohistopathology may have different isoform signatures that require individually adapted anti-neoplastic therapy.

Modes of translation differ in different types of neoplasms. Some switch to translation by neoplasm tic splicing only during proliferation; others, mostly of poor clinical prognosis, periodically turn dysregulated gene-specific ncRNA/mRNA interactions on and off. Still others monitor translation of gene transcriptomes by spontaneous expression of their own disease-associated isoforms without epigenetic triggers (75, 76). For example, carcinogenic isoforms like POLR2K are preferentially upregulated in leukemic and other neoplastic tissues (38, 58). In pulmonary adenocarcinomas, multimeric cis-regulating isoform variants of nicotin receptors modulate missense single-nucleotide polymorphism (CHRNA5) at coding loci. In endometrial adenocarcinogenesis, expression of pathogenetic fibroblast growth factor receptors (FGFRs) is altered by isoforms. In bladder carcinomas and chronic lymphocytic leukemias, neoplastic cell proliferation is promoted by newly acquired replication-dependent mRNA isoform H2A1C and H2A1B/E. (In contrast to canonical H2A, these variants result from mutations in five histone H2A encoding genes at end positions of UTRs 3′ and 5′). In EMT of breast cancer, spliced isoforms including hMENA (a regulator of the actin cytoskeleton expressed in pre-mRNA lacking exon VI) appear in malignant invasive progression. Central coordinators ESRP1, 2, mediating carcinogenic splicing and differentially regulated isoforms such as TPAP2A, may affect tamoxifen therapy.

Clearly, carcinogenic isoform variants of TFs are of key importance in neoplastic translation and phenotypic expression. This complies with the concept of carcinomas being epigenetic diseases (11). It is a matter of speculation whether the final decision for clinical expression of carcinogenic genes also depends on the ability of the genome to “learn” adopting their splicing behavior in epigenetic environmetal networks to altered gene replication patterns (15). As tumor markers, isoforms prove sensitive to specific biochemical therapeutic inhibitors (77, 78). For example, in treatment of colorectal cancers, variant isoforms should be selected.

### Novel genes

*De novo* synthesis of genes has a major impact on evolutionary traits. Indeed, genomes adopt to new epigenetic situations, such as transgenerational information, exposure to environmental carcinogenic contaminants, and other forms of biotic stress that are known to be mutagenic in ES cells (9, 11, 13, 14, 79). Functional alterations are introduced either by gene/protein neofunctionalization (discussed above) or by TFs neosynthesis for selected mutated genes. Translation of the latter is implemented by spliced isoforms that introduce new CpG methylation patterns at sites of DNA “hotspots” (3). Details of selecting TFs from reservoirs of protogene substrates for carcinogenic gene splicing and propagation in mitotic replication forks remain engimatic (5). Materials for DNA neosynthesis are raw, open to modulations by chromatin histones. *MEG-*derived ubiquitin-mediated proteolysins1 (ZAPAC-Ump1) (80) predominantly control the synthesis of novel genes during gamete-to-zygote transitions (MZT) (21, 81). Interestingly, this includes encoding of DNA templates for zygotic TFs-mRNA/ncRNA and proteins that mediate transgenerational transmission of parental memory (82). Also involved are numerous supportive factors, including CCR_4_-NOTs and CTCF. The latter is a multifunctional transcription zinc finger repressor with insulator qualities. Its binding patterns shape DNA–protein interactions for evolving new gene expression. A recent database of CTCF-binding sites is available (83). CCR_4_-NOT complexes are recruited by sequence-specific mRNA-binding proteins including Nanog. They modulate gene translation by deadenylase-catalyzing enzymes, and facilitate decay processes of epigenetic, gene-encoded mRNA-independent allelic poly (A) tails and of co-translational proteasomal proteins. Complexes appear to bind to 3′-UTR-mRNA elements via ncRNA (84). Interestingly, CCR_4_, through its subunit NOT5, connects transcription with translation. Novel TFs identify overlapping domains and modify transcription/translation patterns of parental pre-PGCs and GZES cells to mature to embryonic germ cells (PEGCs) (85). Thus, interactions of parental genes in the zygote reprogram PEGCs as a key source for stem cell memory persisting throughout life (28, 86). Novel genes can also affect splicing of additional RNA isoforms, ­protein–DNA and protein–protein interactions, including tandem repeats of neoplastic mutations (13, 87).

If functionally superior, isoform copies are introduced as new TF-coding genes into the genome for persistent expression in clinical translation and establish carcinogenic genes (3). Thus, novel genes obtained by neofunctionalization and gene neosynthesis individualize the genomic uniqueness of ES cells. Novel genes generate new epigenetic networks of TFs with histone-modified, binding site-specificities that may overlap with other biosynthetic pathways (6, 88). Re-writing of entire genomic programs enables TFs of ES, post-natal, and, to some extent, neoplastic stem cells to respond to environmental stimuli by selecting, targeting, steering of DCSGs (duplication copies of selected genes), and altering epigenetic gene-silencing. Re-writing proceeds synchronously in *GATA1*-regulated complexes driven by context-specific nucleosomes that may determine the clinical outcome of cancer patients (89, 90).

### Pre-neoplastic gene translation

**Figure 2:**
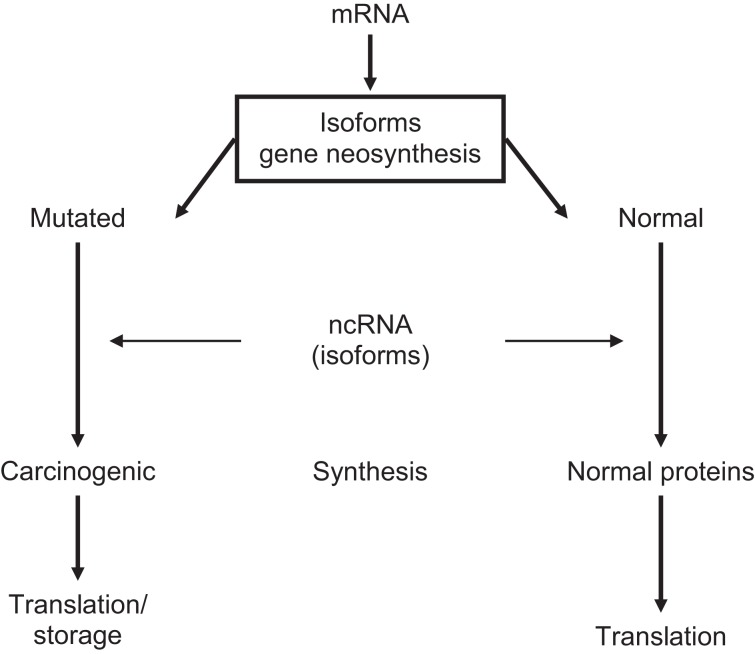
**Neoplastic translation determined by ncRNA-modified mRNA isoforms**.

Carcinogenesis is a slowly developing, multifactorial, highly synchronized process that requires preparatory gene-encoded epigenetic steps. Every cancer has its own neoplastic history that may originate already during embryonic development (13, 91, 92). Pre-neoplastic lesions are shaped at 3′/5′-UTRs from parental isoforms carrying oncogenic memory into abnormal translating DNA templates. It can, therefore, be assumed that cells with pre-neoplastic properties are present in normal ES cell populations. Because gene-encoded isoforms of mRNA/ncRNA TFs catalyze translation of post-transcriptional genes, variants of abnormally spliced tumor-specific TF isoform will determine the expression of carcinogenic/mutated genes. Indeed, through gene neogenesis/neofunctionalization, TFs variants may generate pre-neoplastic subpopulations among ES cells. In addition, carcinogenic isoforms of ppTFs could establish links for interactions with pre-cancerous genomes during zygote-to-GZES cell switches and contribute to decision-making in transgenerational translation of mutated genes (5, 35, 56, 92), presented in Figure 2.

Examples of clinically apparent pre-neoplastic lesions in congenital/childhood acute myeloid/promyeloid leukemias (35, 93) include new AML1-ETO/ERG binding sites, FLI1 demarcating targets of aberrant epigenetic regulation and chromatin accessibility, retinoic acid receptors, and histone acetylation (94–96). It is of great interest to collect more information on the selection of splicing duplication copies for isoforms that induce neoplastic transformation. Research needs to be done with regard to when, and at what occasions in an embryo/patient’s life time, specific carcinogenic lesions are introduced into the genome; how the multitude of crucial metabolic alterations are gene-encoded as adjuvant TFs; how they are synchronized during storage in pre-carcinogenic cells; and whether anti-neoplastic defense mechanisms exist in zygote/GZES cell switches.

## Epigenetics of the Zygote

Forming the zygote by fusion of the two haploid parental pronuclei is a critical event in embryonic development because the diploid pluripotent ES cell genome that controls the entire biological life of the offspring is initiated in the zygote (26). Until fertilization, the two gametes had separate, independent epigenomes each carrying its own transcriptional memory (97). Zygotic fusion changes the situation drastically: now, the one-cell zygote accomodates bi-nucleate transcriptional information from the still separate haploid genomes of both parental pronculei. Zygote formation is initiated through activation of adhesion molecules that bind cis-positioned proteins at oocytic nuclear matrix sites (MARS) to surface membranes of the decondensed spermatocyte (98). Maternal fusogens (of the CD9 tetraspanin TF family) allow the spermatocyte to enter the oocyte through IgSF receptor IZUMO1. Within 3 h of fusion, the spermatocyte terminates maternal meiotic phase II permitting gene programing for the somatic diploid zygote/ES cell genome (99).

Zygote nuclear programing is initiated through pronuclear ZGA by catalyzing TFs that also act as drivers for GZES cell switches (81). It is implemented during the first two cleavages, prior to the first diploid zygotic mitosis at the third, four- to eight-cell division (27, 82), after DNA-CpGs methylation patterns have been altered by chromatin histones, namely proliferation-promoting histones (H3K4/H3K9/H3K18/H3K27), repressive H4K12 acetylation, and H3S10 phosphorylation (21, 24). TFs employ methyltransferases DNMT3a/b/1 and polycomb EZH2 proteins for DNA programing (100). DNMT3A is regulated by DNMT3L and DNMT3b in regulatory feedback loops of mRNA isoforms. Methyl groups from parental DNA are copied by DNMT1 onto *de novo* synthesized daughter strands. Mutant DNMT1s cause loss of DNA methylation, distortion of gene expression, and/or mitotic arrest. Histone deacetylases control mitotic cycle progression. Their inhibitors, histone deacetylase inhibitors (HDACi), have been used in anti-neoplastic therapy (77, 101, 102). Phosphorylation changes affinities of binder proteins to reader/writer modifications that affect crosstalks between histones exerting spatio-temporal controls of chromatin-associated events (103). ppTFs, such as somatic Oct4, Nanog and Sox2, assume special positions in ES cell renewal and in transformation of CSCs. This is separately discussed below in Sections “Zygote-to-GZES cell switches” and “Origin of carcinogenic ppTFs.” Mistakes in interactions of post-fusion pronuclear and zygotic genes lead to severe GZES cell damage, abortions, malformations, and post-natal diseases (46, 104), including mismatches in competitive gene-silencing during intra-S-phase surveillance in leukemogenesis (38).

Imprinted genes also are involved in the generation of diploid GZES cell genomes. They are either reconfirmed by re-imprinting in their former parental DNA-CpG methylation patterns, or are replaced by newly imprinted somatic genes. Imprinting is implemented by selective TFs-mediated CpG methylation. Catalyzing enzymes are TET1, 2 and KDM1B demethylases, and isoforms of DNMT1s and DNMT1o (derived from DNMT1) (105). The paternal genome is re-/imprinted around puberty, independent of fertilization (99). It induces regulatory epigenetic marks for histone packaging and chromatin stabilization in late fetal development (21) in particular in genes *Igf1, 2/Igf2Rs, Peg3, Zim1*, *H19*, and *Zac1* (100, 106). In the maternal pronucleus, re-/imprinting is initiated upon release from meiotic silencing within the first post-fertilization hours preceding ZGA (97). Imprinted genes appear to be particularly vulnerable to distortions of differential marks (49, 99, 106), but are protected by maternal Stella DPPA3s (107) from additional alterations during somatic programing. Abnormally imprinted genes may cause developmental/clinical disorders by promoting survival of pre-cancerous cells among normal ES cell populations (13, 28, 108, 109).

### Zygote-to-GZES cell switches

Transfer experiments have shown that nuclei of end-­differentiated cells, such as mature fibroblasts, can be reprogramed back to full embryonic pluripotency of GZES cells by cytoplasmic TFs from enucleated oocytes (110). Thus, embryonic TFs can establish transcriptional links between GZES and other types of stem cells, including pre-/neoplastic cells (13, 58, 111). In fact, gametes transmit to the zygote genomic inheritable information on parental neoplastic memory for processing during generation of GZES cell genomes, along with normal information (83, 112). Signaling programs sharing information on transformation of self-maintenance and migration are conveyed to both ES cells and CSC, for example by reversible EMTs. They are driven by TFs SNAIL/TWIST and ZEB (7, 8) and completed by *de novo* synthesis of embryonic TF isoforms (12, 92). Linking embryonic to neoplastic transcription is mediated by common, or closely related, ppTFs such as Nanog, Oct4, and Sox2. ppTFs that operate in regulatory signaling pathways of the cell cycle of both GZES and CSC are hallmarks of unlimited proliferation and self-renewal. They synergize with one another, with other types of isoforms, and with lncRNA as scaffolds for chromatin-modifying complexes (4, 113, 114). ppTFs are encoded by genes *WNT/*β*-catenin* (115, 116), *JAK/STAT* (117, 118), *NOTCH* (119), *MAPK/ERK* (120), and *PI3K/AKT* (121). *JAK* (Janus activating tyrosine kinase) triggers the Wnt/β-catenin pathway (encoded by gene *Ctnnb1*) as tumorigenic gatekeeper in GZES cells for myeloproliferative diseases (116, 118, 122). The *Notch* pathway controls intercellular communication. *MAPK/ERK* are mitogen-activated/extracellular signaling pathways. *PI3K* is a phosphoinositide-3-protein kinase.

*Oct4* is a master gene for pluripotency and self-renewal of undifferentiated GZES cells (123). It is expressed in oocytes, PGCs, and the embryonic inner cell mass (ICM) of pre-implantation blastocysts (124). Oct4 operates through ubiquitin E3 ligase Itch that is involved in self-renewal, pluripotency induction, somatic cell reprograming, and protein stability of transcribing ES cells (125). In cultures of heterologous Oct4^±^ ES cells, Oct4 strongly binds to key regulatory chromatin histones. This increases *Wnt* signaling for enhanced cell sensitivity to LIF (leukemia inhibitory factor), reinforces network pluripotency, and promotes resistance to differentiation. Switches to homologous TF expression (Oct4^+/+^) induce GZES cell differentiation (4). Oct4 also is a key determinant of gene expression by CSCs. Although it acts as a general neoplastic TF in EMT-mediated stemness signatures, it may not be a specific neoplastic driver (123, 126). Oct4 in p53-miRNA-34a/p63 miRNA-34a loops (127, 128) affects leukemogenic signaling pathways through balances between p63 stimulation and p53 downregulation (129). Other pathways such as *Lin28/Oct4* in ovarian cancer (130), carry Oct4 as a promotor bound to cyclin D1 (CCND1) protein. A data base is available on integrated retrival of Oct4 related in human and mouse ES, embryonic cancer cells (ECC), and CSCs (131).

Pluripotent TFs Nanog programs and maintains pluripotency of naïve ES cells at the target locus *Esrrb* synergizing with Oct4–Sox2 binding motifs (132). Nanog levels in the ICM are stimulated by adenylate cyclase and are downregulated in *JAK/STAT* pathways (133). In complexes with 5mC hydroxylases TET1/2, Nanog determines cell fate and pluripotency of ES cells and CSCs (131, 134) and implements tissue specificities of genes *Brca1* (modulator of cellular stress/repair), *Trp53* (tumor repressor), and *Rb1* (retinoblastoma suppressor) (135, 136). Overexpressed Nanog may act as driving factor in cell proliferation and self-renewal of human and murine neoplasms by controlling molecular stemness in *Wnt/*β** and *Tcf3/Tcf1*β*-catenin* signaling pathways (137).

Sox2 protein is involved in pre-implantation development. It is expressed in 16-cell bovine embryos and is restricted to the ICM. Sox2 operates in negative GZES cell feedback loops of *Akt* signaling and binds to Fox01, a nuclear TF in metabolic and energy homeostasis (138). Increases of Akt serine/threonine kinase cause decreases in endogenous Sox2 in association with losses of Fox01 (121, 127, 139). In metastasizing neoplasms, Sox2 stimulates signaling pathways *Wnt*β*-catenin* EMT (140).

### Origin of carcinogenic ppTFs

**Figure 3:**
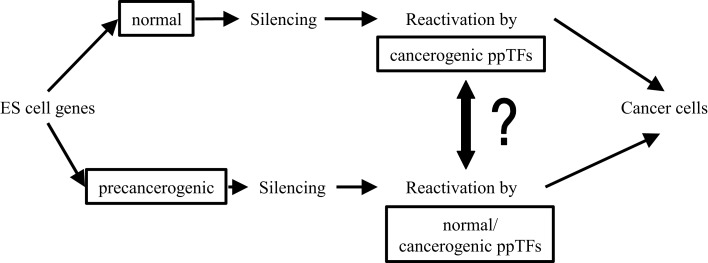
**Links between normal embryonic and pre-neoplastic transcription factors (TFs)**.

Although, clearly, ppTFs are indispensible drivers for pluripotent proliferation/self-renewal in both ES and neoplastic cell transformation, the origin of carcinogenic ppTFs in CSCs is still elusive. In Figure 3, questions of links between normal embryonic and carcinogenic ppTFs are raised: are they biochemically identical or can they be distinguished from one another? Do TF isoforms acquire carcinogenic propeties during splicing and, if so, how? Are carcinogenic ppTFs derived from normal ppTFs or are they generated sui generis? What triggers functional alterations? Are pre-neoplastic ES cells activated by both, normal and neoplastic ppTFs? Or are the latter generated through TF gene mutations from primordial germ cells, through reactivation of silenced embryonal TFs, or through neosynthesis via mRNA/ncRNA spliced isoforms? What functions of ppTFs are reversible in EMT/MET? A recent study suggests on the basis of molecular differences in functionally separable ppTFs that neoplastic ppTFs are not reactivated from silenced ES cells, but rather are distinct regulators in newly composed epigenetic networks (141). So, it remains an unsettled question how genomic interactions of ppTFs link embryonic/normal with neoplastic development (142–147).

## Epigenetic Anti-Neoplastic Therapy

Therapeutic enzymatic reprograming of the genome aims to alter the developmental fate of CSCs. It is hoped to abolish or mitigate carcinogenic cell properties in favor of normal gene functioning by reversing transcriptional signatures. Potential therapeutic targets are transcription-driving molecules in transport, binding of nuclear/cytoplasmic receptors, and drug-metabolization. Enzyme inhibitors focus on neoplasm-specific TFs of gene imprinting, mono-allelic transcription, post-transcriptional gene translations, and ZGS (78).

Promising attempts of interfering with enzymatic reactions have so far mostly been limited to *in vitro* cell lines, but clinical studies are in progress. Drugs include decitabine (5-ara-2′-deoxycitidine), HDACi, H3 histone acetylator of tumor-suppressor gene *PRDX2*, and polo-like kinase SNK/PLK2 (a transcriptional target for wild-type p53). Other approaches have re-enforced tumor suppressor genes by inhibition of lysine-specific LSD1 demethylase for H3K4me1, 2 in acute myelogenous leukemia and small cell lung cancer by activation of all-retinoic-acid differentiation pathways, or have altered DNMT-mediated DNA-CpG methylation patterns (1, 94). Indeed, carcinomatous cell growth should be classified according to its sensitivity to drug-inhibiting key enzymes.

## Summary

**Figure 4:**
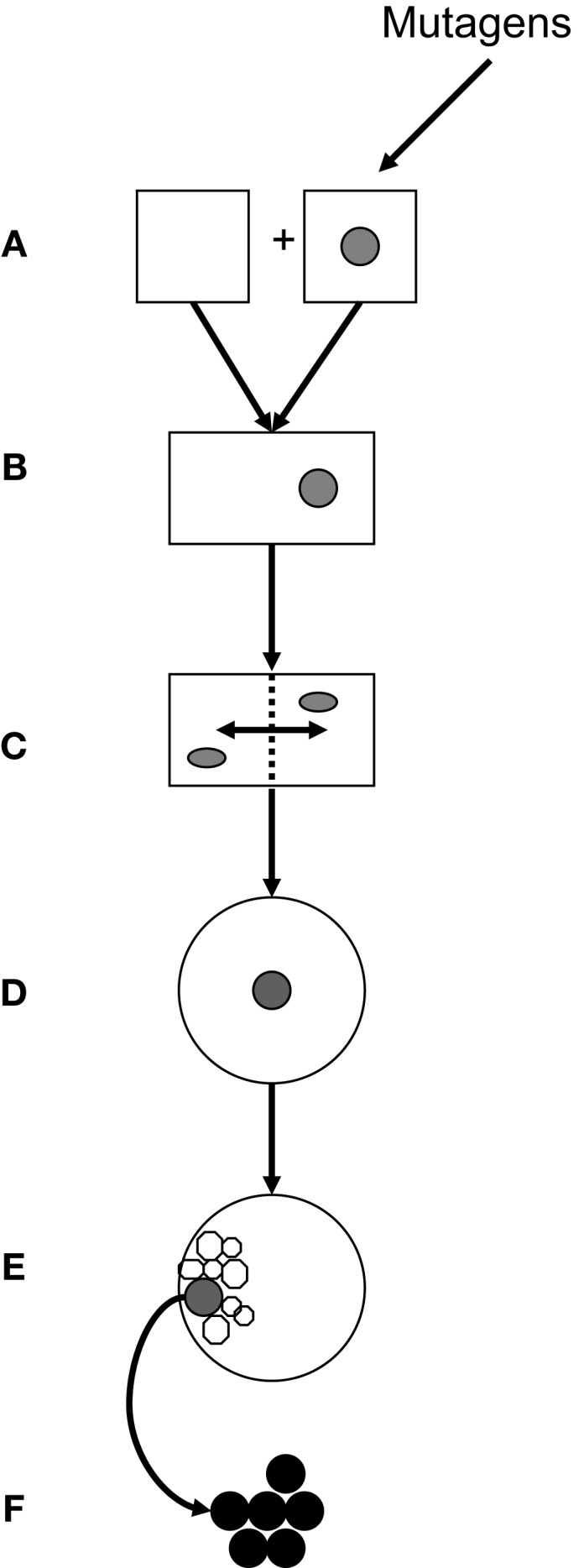
**Transgenerational transmission of carcinogenic gene lesions**. **(A)** Parental gametes; one being hit, e.g., by an environmental insult (mutagen). **(B)** Fertilization, fusion of gametes, and formation of the zygote. **(C)** Interactions during the first two zygote cleavages of post-fusion parental genes, including genes carrying pre-neoplastic DNA damage, are modulated by chromatin histones. **(D)** Genes with pre-neoplastic DNA lesions participate in zygote-to-ES cell switches **(E)** Pre-neoplastic genes are included in the internal cell mass. **(F)** Overt carcinogenesis.

Genetics and epigenetics constitute a functional entity in embryonic and post-natal cell proliferation. This review focuses on potential gene interactions in cell cycles of gametes, zygotes, and ES cells that may be related to neoplastic transformation. Figure 4 presents steps of transgenerational transmission of carcinogenic information: Carcinogenic epigenetics may be initiated during pre/conception and gestation through environmental exposure to mutagens (Figure 4A). Exposures may cause abnormal exchanges between recombining alleles during parental meiosis or during pronuclear interactions in the zygote, and generate abnormally spliced TFs (mRNA/ncRNA) isoforms that are transmitted to GZES cells. Isoforms may induce gene neosynthesis and neofunctionalization of silenced pre-existing (ancestral) TFs genes. Carcinogenic pre-/information can be caused by TFs duplication copies that deviate biochemically/functionally from their originals. Abnormal information is transmitted to the zygote, participates in nuclear programing, and is incorporated in the zygotic genome (Figure 4B). Early transgenerational transmission by ZGA is dominated by maternal effect genes *MEGs* in both pronuclei. Pre-/neoplastic DNA damage is modulated by chromatin histone H3 methylation/acetylation and interacts with its zygotic fusion partner (Figure 4C), predominantly during the first two zygote cleavages. This establishes parental oncogenic memory in the zygote. Pre-neoplastic DNA lesions participate in ZGS mediated by abnormally spliced carcinogenic TFs isoforms (Figure 4D), and are included in the ICM of the embryo (Figure 4E). Clearly, further research needs to be done on carcinogenic splicing of TFs isoforms.

In clinically overt carcinogenesis (Figure 4F), carcinogenic TF isoforms are organized in epigenetic networks alongside normal TFs, operating through specific binding sites. Carcinogenic transgenerational inheritance appears to be connected to normal GZES cell development by signaling links provided by ppTFs, such as Oct4, Nanog, and Sox2. In fact, ppTFs convey proliferative, self-maintaining, invasive/migratory genomic programs to both normal and pre-neoplastic daughter cells. Whether ppTFs are biochemically identical in both instances is unknown. Pre-carcinogenic transcriptional information is storaged in heterochromatin waiting for post-natal triggering by additional stimuli in synergy with full support from adjuvant metabolic TFs. Possibly, equilibria between ppTFs of normal and carcinogenic translation could be pushed therapeutically in favor of normal translation. Thus, TFs are cell/disease-specific messengers that may trigger the expression of clinically overt neoplasms by deciding on post-natal carcinogenic gene translation. Accordingly, carcinomas have recently been defined as “epigenetic diseases.”

## Conflict of Interest Statement

The author declares that the research was conducted in the absence of any commercial or financial relationships that could be construed as a potential conflict of interest.

## Appendix

### Abbreviations

5hmc, 5hydroxy-methyl-cytosine; 5mc, 5methyl-cytosine; A-to-I, adenosine-to-inosine; AID, autoinflammatory disease proteins; ALL, acute lymphoblastic leukemia; AML, acute myelogenous leukemia; AML-ETO, t(8;21)AML; APA, alternative polyadenylation; Blimp: transcription repressor of somatic Hox and Snail genes; BRCA1: modulator of cellular stress and DNA repair; with a protective role in autophagy in tumorigenesis/prognosis; C2-WW-HECT: Smad ubiquitylation (Smurf1) domain E3; mediated by a protein–protein WW linker; CBFβ: transcription cofactor to the ubiquitin ligase complex; CCND1: cyclin D1 has an essential role in cancer progression; CCR4-NOT: a multi-subunit de-amylase complex that regulates gene expression; CD44, cell membrane glycoprotein, alternatively spliced exon; Cdh1, e-cadherin; C/EBP, CCAAT enhancer binding protein; CHRNA5, missense coding single-nucleotide polymorphism (SNP); CLL, chronic lymphatic leukemia; CPE, cytoplasmic polyadenylation element; CPEB, binding proteins; CpG, cytosine phosphate guanine; CPCF, catalyzation specificity factor; CPSF, polyadenylation specificity factor; CSC, cancer stem cell; CTCF, transcription repressor CCCTC zinc finger protein for gene regulation; Ctmb, cellulose tris (4-methylbenzoate), a cubic transport model base; DACHS, a cell fate factor (Dachshund) that mediates oncogenic transcription and translation by suppressing EMT; DAZL, deleted-azoospermia-like; Dbp2, RNA-dependent helicase; Dbp5, mRNA export factor in ATP dependent remodeling of RNA/protein complexes; DCSG, duplication copies of selected genes; DEAD, Dbp5 nucleo-cytoplasmic proteins; DM, DNA demethylases; DNMT, DNA methyltransferase; Dppa, periplasmic polypeptide haloalkane dehydrogenase (maternal factor Stella); e1F4H1, spliced translation-initiation e1mRNA; ECCs, embryonic cancer cells; eeRNA, endogenous enhancer RNA; eEF1A1 isoform, translation elongation factor; EMT/MET, epithelial-mesenchymal/mesenchymal-epithelial transition; ERG, FL1, closely related members of the; ETS family of transcription factors; ES, embryonic stem (cells); ESRP, central coordinator; Ezh, enhancer of zeste homologe; FGFR, fibroblast growth factor receptor; Fip1, mRNA3 processing factor essential for ES cell self-renewal and somatic cell reprograming; Fip1/1, gene encoding Fip1; FL1, transcription factor; Foxo1, nuclear transcription factor, especially in metabolism and energy homeostasis; Gadd45, DNA damage-inducible45 protein; GATA, transcriptional regulator proteins; GSF–MEG, gonad-specific factor–maternal effect genes; GW182, key regulator of neuropeptide PDF signaling; GZES, gamete-to-zygote-embryonic stem (cells); H1(0), linker histones; H2A1B/E, H2A1C, replication dependent isoforms; H3K, histone 3 kinase; HDACi, inhibitors of histone deacetylase control; hMENA, human regulator of the actin cytoskeleton; HOTAIRM1, encoded in the human HOXA gene cluster; HOXA, HOXA genes are regulators of normal and malignant hematopoiesis; Hsf, heat shock; ICM, internal cell mass of the early embryo; ICR, cis-reacting imprinting control regions; Igf, insulin-like growth factor; IgfR, insulin-like growth factor receptor; IgSF, immunoglobulin superfamily that inhibits adhesion to endothelial inflammatory ligands; Itch, E3 ligase; Izumoi, oocyte IgSF receptors for sperm entry; JAK, Janus activating tyrosine kinase; JAK/STAT, stem cell regulating signaling pathway. Misregulations can be involved in cancer; KDM1B, a H3K4 demethylase for maternal genomic imprints; LIF, leukemia inhibitory factor; lncRNA, long ncRNA; LysRS, lysyl-tRNA synthetase that inactivates translational functions in favor of transcription through factor MITF; MAPK/ERK, mitogen-activated protein kinases/extracellular-signal-regulated kinases; Key regulators of cancer hallmarks; MARS, matrix attachment site; MBD4, methyl-CpG binding domain 4; MDS, myelodysplastic syndrome; MEGs, maternal effect genes; MEK, includes seven kinases that participate in the RAS/RAF/MEK/ERK signal transduction pathway; MIL1, microsporeless1, a high-affinity blocker of mouse IL1alpha/beta; MLL, mixed lineage leukemia gene; mRNP, ribonucleoprotein; Myc, oncoprotein, TF that regulates the expression of many genes involved in cell growth, proliferation, and metabolic pathways; MZT, maternal-to-zygote transition; Nanog, homeodomain ppTF of ES cells; ncRNA, non-coding RNA; Nlrp, early zygote blastomere apoptosis protei; Notch, intercellular communication pathway with key roles in congenital/neoplastic diseases; Npm, nucleoplasmic; Oct4, a pp TF; PABP1, poly A-binding, ubiquitous, multifunctional MRNA1 protein; PAN2/3, poly(A) binding proteins 2,3; Peg3, imprinted regulating gene domain e.g., for fetal growth and maternal caring behavior; PEGS, primordial embryonic germ cells; PGCs, primordial germline cells; PI3K/AKT, phospho-inositide-3-protein kinase; Piwi, protein of the ARGONAUTE family; piRNA, piwi, retrovirus interacting transposon; PML, promyelocytic leukemia; Pms2, mismatch repair gene2 mutation; Pol, polymerase; POLR2K, isoform, preferentially upregulated in cancer cells; POUF, homeobox gene Pit-1 encoded TF; ppTFs, pluripotent TFs; Rb1, retinoblastoma suppressor gene; RBP, RNA binding protein; RBS, ribosome binding site; RGG, motif boxes rich in argenine and glycine; RRM, RNA regulating motif; RUNX, mRNA kinase, hematopoietic TFs; SCLC, small cell lung cancer; SCMC, subcortical maternal complexes; SIR, silent information regulator; siRNA, small interfering ncRNA; sRNA, small, ncRNA; Sca1, CD133, CD34, ALDH1, CSC markers; Smarca4, Brahma related gene1 (Brg1); Snail, transcriptional factor for cell invasion, that downregulates E-cadherin and upregulates MMP; SNK/PLK, serum-inducible, polio-like kinase, a tumor suppressor in B-cell malignancies; Sox2, a ppTF, embryonic stem cell marker; SPI1, key gene for hematopoietic regulation and leukemia suppression; TAL1, hematopoietic regulator; Tcf, T-cell factor; TCS, translational control sequence; TFBS, TF binding site; TPAP2A, differentially regulated isoform; TSS, TF start site; TET, ten eleven translocation protein; TF, transcription factor; TRNA, transfer RNA; Trp53, tumor repressor protein; TWIST, basic helix-loop-helix transcription factor that promotes cancer metastases; UTR, untranslated region; VEGF, vascular endothelial growth factor; Wntβ-catenin, signaling pathway in embryonic and adult human stem cells. Deregulations can be involved in developmental and genetic diseases including cancer; Zac1, zinc finger protein1 that regulates apoptosis; ZAPAC-Ump, maternally derived ubiquitin-mediated proteolysin; ZAR, zygote arrest; ZARL, ZAR-like; ZBE, Zinc finger binding element and transcriptional activator of Znf131 involving; sequence-specific Kaiso binding sites; Kaiso is a negative regulator of the Wnt/β-catenin TCF signaling pathway; ZEB, family of transcription factors in embryonic development that also induce EMT in cancer invasiveness and CSC stemness; ZGA, zygote gene activation; ZGS, zygote-to-GZES cell switches; Zim1, maternally expressed trans factor controled by paternal Peg3.

## References

[1] Jones PA. Functions of DNA methlation: islands, start sites, gene bodies and beyond. Nat Rev Genet (2012) 13:1–9. 10.1038/nrg3230 22641018

[2] Lamm E. The genome as a developmental organ. J Physiol (2014) 592(11):2283–93. 10.1113/jphysiol.2014.271734 24882813 PMC4048088

[3] Qian W Zhang J. Genomic evidence for adaptation by gene duplication. Genome Res (2014) 24(8):1356–62. 10.1101/gr.172098.114 24904045 PMC4120088

[4] Karawacki-Neisius V Göke J Osorno R Halbritter F Ng JH Weiße AY Reduced Oct4 expression directs a robust pluripotent state with distinct signalling activity and increased enhancer occupancy by Oct4 and Nanog. Cell Stem Cell (2013) 12(5):531–45. 10.1016/j.stem.2013.04.023 23642364 PMC3650585

[5] Leman AR Noguchi E. The replication fork:understanding the eukaryotic replication machinery and the challenges to genome replication. Genes (2013) 4(1):1–32. 10.3390/genes4010001 23599899 PMC3627427

[6] Sato F Tsuchiya S Meltzer SJ Shimibu K. MicroRNA and epigenetics. FEBS J (2011) 278(10):1598–609. 10.1111/j.1742-4658.2011.08089 21395977

[7] Brown RL Reinke LM Damerow MS Perez D Chodosh LA Yang J CD44 splice isoform switching in human and mouse epithelium is essential for epithelial-mesenchymal transition and breast cancer progression. J Clin Invest (2011) 121:1064–74. 10.1172/JCI44540 21393860 PMC3049398

[8] Caramel J Papadogeorgakis E Hill L Brown GJ Richard G Wierinckx A A switch in the expression of embryonic EMT-inducers drives the development of malignant melanoma. Cancer Cell (2013) 24:466–80. 10.1016/j.ccr.2013.08.018 24075834

[9] Manikkam M Haque MM Guerrero-Bosagna C Nlisson EE Skinner ML. Pesticide methoxychlor promotes the epigenetic transgenerational inheritance of adult-onset disease through the female germline. PloS One (2014) 9(7):e102091. 10.1371/journal.pone.0102091 25057798 PMC4109920

[10] Kumar S Saradhi M Chaturvedi NK Tyagi RK. Retention and transmission of active transcription memory from progenitor to progeny cells via ligand-modulated transcription factors: elucidation of a concept by BIOPIT model. Cell Biol Int (2012) 36(2):177–82. 10.1042/CBI20090329 22007870

[11] Burgio E Migliore L. Towards a systemic paradigm in carcinogenesis: linking epigenetics and genetics. Mol Biol Rep (2015) 42(4):777–90. 10.1007/s11033-014-3804-3 25387435

[12] Wu PYJ Nurse P. Replication origin selection regulates the distribution of meiotic recombination. Mol Cell (2014) 53(4):655–62. 10.1016/j.molcel.2014.01.022 24560273 PMC3988929

[13] Marshall GM Carter DR Cheung BB Liu T Mateos MK Meyerowitz JG The prenatal origins of cancer. Nat Rev Cancer (2014) 14:277–89. 10.1038/nrc3679 24599217 PMC4041218

[14] Skinner MK Haque CGBM Nilson E Bhandari R Carrey JR. Environmentally induced transgenerational epigenetic reprogramming of primordial germ cells and the subsequent germ line. PloS One (2013) 8(7):e66318. 10.1371/journal.pone.0066318 23869203 PMC3712023

[15] Miligi L Bevenuti A Mattioli S Salvan A Tozzi GA Ranucci A Risk of childhood leukemia and non-hogkin’s lymphoma after parental exposure to solvents and other agents: the SETL Study. Occup Environ Med (2013) 70:648–55. 10.1136/oemed-2012-100951 23729503

[16] Booth MJ Branco MR Fiez G Oxley D Krueger F Reik W Quantitative sequencing of 5-methylcytosine and 5-hydroxymethylcytosine at single base resolution. Science (2012) 336:934–7. 10.1126/science.1220671 22539555

[17] Iqbal K Jin SG Pfeifer GP Szabo PE. Reprogramming of the paternal genome upon fertilization involves genome-wide oxidation of 5-methylcytosine. Proc Natl Acad Sci U S A (2011) 108(9):3642–7. 10.1073/pnas.1014033108 21321204 PMC3048122

[18] Hashimoto H Liu Y Upadhyay AK Chang Y Howerton SB Vertino PM Recognition and potential mechanisms for replication and erasure of cytosine hydroxymethylation. Nucleic Acids Res (2012) 40(11):4841–9. 10.1093/nar/gks155 22362737 PMC3367191

[19] Hatanaka Y Shimizu N Nishikawa S Tokoro M Shin SW Nishihara T GSE is a maternal factor involved in active DNA demethylation in zygotes. PloS One (2013) 8(4):e60205. 10.1371/journal.pone.0060205 23560077 PMC3613368

[20] Park MW Kim KH Kim EY Lee SY Ko JJ Lee KA. Associations among *Sebox* and other MEGs and its effects on early embryogenesis. PLoS One (2015) 10(2):E0115050. 10.1371/journal.phone.0115050 25679966 PMC4331730

[21] Ostrup O Andersen IS Collas P. Chromatin-linked determinants of zygotic genome activation. Cell Mol Life Sci (2013) 70(8):1425–37. 10.1007/s00018-012-1143-x 22965566 PMC11113722

[22] Sugito K Kawashima H Yoshizawa S Uekusa S Hoshi R Furuya T Non-promotor DNA hypermethylation of zygote arrest 1 (ZAR1) in neuroblastoma. J Pediatr Surg (2013) 48(4):782–8. 10.1016/j.jpedsurg.2012.08.008 23583134

[23] Di Stefano M Rosa A Belcastro V di Bernardo D Micheletti C. Colocalization of coregulated genes: a steered molecular dynamics study of chromosome 19. PloS Comput Biol (2013) 9(3):e1003019. 10.1371/journal.pcbi.1003019 23555238 PMC3610629

[24] Chen WC Wu PH Phillip JM Khatau SB Choi JM Dallas MR Functional interplay between the cell cycle and cell phenotypes. Integrat Biol (2013) 5(3):523–34. 10.1039/c2ib20246h 23319145 PMC3813296

[25] He J Stewart K Kinnele HL Anderson RA Childs AJ A developmental stage-specific switch from DAZL to BOLL occurs during oogenesis in humans, but not mice. PloS One (2013) 8(9):e73996 10.1371/journal.pone.0073996 24086306 PMC3783425

[26] Seisenberger S Peat JR Hore TA Santos F Dean W Reik W. Reprogramming DNA methylation in the mammalian life cycle: building and breaking epigenetic barriers. Philos Trans R Soc London B Biol Sci (2013) 368(1609):20110330. 10.1098/rstb.2011.0330 23166394 PMC3539359

[27] Shin SW Shimizu N Tokoro M Nishikawa S Hatanaka Y Anzai M Mouse zygote-specific proteasome assembly chaperone is important for maternal gamete-to-zygote transition. Biol Open (2013) 2(2):170–82. 10.1242/bio.20123020 23429752 PMC3575651

[28] Pirouz M Klimke A Kessel M. The reciprocal relationship between primordial germ cells and pluripotent stem cells. J Mol Med (2012) 90(7):753–61. 10.1007/s00109-012-0912-1 22584374

[29] Ozgen H Kahya N Jonge de JC Smith GST Harouz G Hoekstra D Regulation of cell proliferation by nucleocytoplasmic dynamics of postnatal and embryonic exon-II-containing MBP isoforms. Biochim Biophys Acta (2014) 1843(3):517–30. 10.1016/j.bbamcr.2013.11.026 24321769

[30] Haldane A Manhart M Morozov AV. Biophysical fitness landscapes for factor binding sites. PloS Comp Biol (2014) 10(7):e1003683. 10.1371/journal.pcbi.1003683 25010228 PMC4091707

[31] Wu DD Irwin DM Zhang YP. De novo origin of human protein-coding genes. PloS Genet (2011) 11:e1002379. 10.1371/journal.pgen.1002379 22102831 PMC3213175

[32] Magadum S Banerjee U Murugan P Gangapur D Ravikesavan R Gene duplication as a major force in evolution. J Genet (2013) 92(1):155–61. 10.1007/s12041-013-0212-8 23640422

[33] Liu H Yin J Xiao M Gao C Mason AS Zhao Z Characterization and evolution of 5’ and 3’untranslated regions in eukaryocytes. Gene (2012) 507(2):106–11. 10.1016/j.gene.2012.07.034 22846368

[34] Zhao W Pollack JL Blagev DP Zaitlen N McManus MT Erle DJ. Massively parallel functional annotation of 3’untranslated regions. Nat Biotechnol (2014) 32(4):387–91. 10.1038/nbt.2851 24633241 PMC3981918

[35] Horsthemke B. Mechanisms of imprint regulation. Am J Med Genet (2010) 154C(3):321–8. 10.1002/ajmg.c.30269 20803654

[36] Shkreta L Bell B Revil T Venables JP Prinos P Elela SA Cancer-associated perturbations in alternative pre-messenger RNA splicing. Cancer Treat Res (2013) 158:41–94. 10.1007/978-3-642-31659-3_3 24222354

[37] Johnson A Wu R Peetz M Gygi SP Moazed D. Heterochromatic gene silencing by activator interference and a transcription elongation barrier. J Biol Chem (2013) 288(40):28771–82. 10.1074/jbc.M113.460071 23940036 PMC3789973

[38] Prindull G. Epigenetic mismatches with mutated transcribing genes at leukemogenic S-phase binding/start sites. Potential targets for therapy with enzyme inhibitors. Curr Stem Cell Res Ther (2012) 7:420–9. 10.2174/157488812804484602 23072459

[39] Beretta S Bonizzoni P Della Vedova G Pirola Y Rizzi R. Modeling alternative splicing variants from RNA-Seq data with isoform graphs. J Comput Biol (2014) 21(1):16–40. 10.1089/cmb.2013.0112 24200390 PMC3880078

[40] Baierlein C Hackmann A Gross T Henker L Hinz F Krebber H. Monosome forming during translation initiation requires the serine/arginine-rich protein Np13. Mol Cell Biol (2013) 33(24):4811–23. 10.1128/MCB.00873-13 24100011 PMC3889561

[41] Ray D Kazan H Cook KB Weirauch MT Najafabadi HS Li X A compendium of RNA binding motifs for decoding gene regulation. Nature (2013) 499:172–7. 10.1038/nature12311 23846655 PMC3929597

[42] Lenasi T Barboric M. Mutual relationships between transcription and pre-mRNA processing in the synthesis of mRNA. Wiley Interdiscip Rev RNA (2013) 4(2):139–54. 10.1002/wrna.1148 23184646

[43] Pre´vost K Desnoyers G Jacques JF Lavoie F Massé E. Small RNA-induced mRNA degradation achieved through both translation block and activated cleavage. Genes Dev (2011) 25(4):385–96. 10.1101/gad.2001711 21289064 PMC3042161

[44] Tieg B Krebber H. Dbp5 – from nuclear export to translation. Biochim Biophys Acta (2013) 1829(8):791–8. 10.1016/j.bbagrm.2012.10.010 23128325

[45] Huang B Zhang R. Regulatory non-coding RNAs: revolutionizing the RNA world. Mol Biol Rep (2014) 41(6):3915–23. 10.1007/s11033-014-3259-6 24549720

[46] Esteller M. Non-coding RNAs in human disease. Nat Rev Genet (2011) 12:861–74. 10.1038/nrg3074 22094949

[47] Xie C Yuan J Li H Li M Zhao G Bu D NONCODEv4: exploring the world of long non-coding RNA genes. Nucleic Acids Res (2014) 42:D98–103. 10.1093/nar/gkt1222 24285305 PMC3965073

[48] Tay Y Kats L Salmena L Weiss D Tan SM Ala U Coding-independent regulation of the tumor suppressor PTEN by competing endogenous mRNA. Cell (2011) 147(2):344–57. 10.1016/j.cell.2011.09.029 22000013 PMC3235920

[49] Gregg C Zhang J Weissbourd B Luo S Schroth GP Haig D High-resolution analysis of parent-of-origin allelic expression in the mouse brain. Science (2010) 329:643–8. 10.1126/science.1190830 20616232 PMC3005244

[50] Gromak N. Intronic microRNA: a crossroad in gene regulation. Biochem Soc Trans (2012) 40:759–61. 10.1042/BST20120023 22817729

[51] Weinberg MS Morris KV. Long non-coding RNA targeting and transcriptional de-repression. Nucleic Acid Ther (2013) 23(1):9–14. 10.1089/nat.2012.0412 23391414 PMC3569965

[52] Cook MC Blelloch R Small RNAs in germline development. Curr Top Dev Biol (2013) 102:159–205. 10.1016/B978-0-12-416024-8.00006-4 23287033

[53] Beck D Ayers S Wen J Brandl MB Pham TD Webb P Integrative analysis of the next generation sequencing for small non-coding RNAs and transcriptional regulation in myelodysplastic syndromes. BMC Med Genomics (2011) 4:19. 10.1186/1755-8794-4-19 21342535 PMC3060843

[54] Ng SY Stanton LW. Long non-coding RNAs in stem cell pluripotency. Wiley Interdiscipl Rev RNA (2013) 4(1):121–8. 10.1002/wrna.1146 23139157

[55] Garitano-Trojaola A Agirre X Prósper F Fortes P. Long non-coding RNAs in haematological malignancies. Int J Mol Sci (2013) 14(8):15386–422. 10.3390/ijms140815386 23887658 PMC3759866

[56] Bolisetty MT Beemon KL. Splicing of internal large exon is defined by novel cis-acting sequence elements. Nucleic Acids Res (2012) 40(18):9244–54. 10.1093/nar/gks652 22790982 PMC3467050

[57] Villalba A Coll O Gebauer F. Cytoplasmic polyadenylation and translational control. Curr Opin Genet Dev (2011) 21(4):452–7. 10.1016/j.gde.2011.04.006 21536428

[58] Lin Y Li Z Ozsolak F Kim SW Sang W Arango-Argoty G An indepth map of polyadenylation sites in cancer. Nucleic Acids Res (2012) 40(17):8460–71. 10.1093/nar/gks637 22753024 PMC3458571

[59] Denti MA Viero G Provenzani A Quattrone A Macchi P. mRNA fate: life and death of the mRNA in the cytoplasm. RNA Biol (2013) 10(3):360–6. 10.4161/ma.23770 23466755 PMC3672278

[60] Wagner SD Yakovchuk P Gilman B Poniscan SL Drullinger LF Kugel JF RNA polymerase II acts as an RNA-dependent RNA polymerase to extend and destabilize a non-coding RNA. EMBO J (2013) 32(6):781–90. 10.1038/emboj.2013.18 23395899 PMC3604716

[61] Renaud M Viviane P Vieu E Florens L Washburn MP l’Hóte P Gene duplication and neofunctionalization: POLR3G and POLR3GL. Genome Res (2014) 24(1):37–51. 10.1101/gr.161570.113 24107381 PMC3875860

[62] Biselli-Chiote PM Oliveira ARCP. VEGF gene alternative splicing: pro- and anti-angiogenic isoforms in cancer. J Cancer Res Clin Oncol (2012) 138(3):363–70. 10.1007/s00432-011-1073-2 22045472 PMC11824452

[63] Kelemen O Convertini P Zhang Z Wen Y Shen M Falaleeva M Function of alternative splicing. Gene (2013) 514(1):1–30. 22909801 10.1016/j.gene.2012.07.083 PMC5632952

[64] Rodriguez JM Marietta P Ezkurdia I Pietrelli A Wesselink JJ Valencia A APPRIS: annotation of principle and alternative splice isoforms. Nucleic Acids Res (2013) 41(Database issue):D110–7. 10.1093/nar/gks1058 23161672 PMC3531113

[65] Cloutier SC Ma WK Nguyen LT Tran EJ. The dead-box RNA helicase Dbp2 connects RNA quality control with repression of aberrant transcription. J Biol Chem (2012) 287(31):26155–66. 10.1074/jbc.M112.383075 22679025 PMC3406699

[66] Nisole S Maroui MA Mascle XH Aubry M Chelbi-Alix M. Differential roles of PML isoforms. Front Oncol (2013) 3:125. 10.3389/fonc.2013.00125 23734343 PMC3660695

[67] Biddle A Gammon L Fazil B Mackenzie IC. CD44 staining of cancer stem cell-like cells is influenced by down-regulation of CD44 variant isoforms and up-regulation of the standard CD44 isoform in the population of cells that have undergone epithelial-to-mesenchymal transition. PloS One (2013) 8(2):e57314. 10.1371/journal.pone.0057314 23437366 PMC3577706

[68] Bergholz J Xiao ZX. Role of p63 in development, tumorigenesis and cancer progression. Cancer Microenviron (2012) 5(3):311–22. 10.1007/s12307-012-0116-9 22847008 PMC3460051

[69] Suzuki H Maruyama R Yamamoto E Kai M. Epigenetic alteration and microRNA dysregulation in cancer. Front Genet (2013) 4:258. 10.3389/fgene.2013.00258 24348513 PMC3847369

[70] Zheng T Wang J Zhao Y Zhang C Lin M Wang X Spliced MDM2 isoforms promote mutant p53 accumulation and gain-of-function in tumorigenesis. Nat Commun (2013) 4:2996. 10.1038/ncomms3996 24356649 PMC3960723

[71] Prange KHM Singh AA Martens JHA. The genome-wide molecular signature of transcription factors in leukemia. Exp Hematol (2014) 42(8):637–50. 10.1016/j.exphem.2014.04.012 24814246

[72] Diffner E Beck D Gudgin E Thomas JAI Knezevic K Pridans C Activity of a heptad of transcription factors is associated with stem cell programs and clinical outcome in acute myeloid leukemia. Blood (2013) 121(12):2289–300. 10.1182/blood-2012-07-446120 23327922

[73] Wu D Matsushita K Matsubara H Nomura F Tomonaga T. An alternative splicing isoform of eukaryotic initiation factor 4H promotes tumorigenesis in vivo and is a potential therapeutic target for human cancer. Int J Cancer (2011) 128(5):1018–30. 10.1002/ijc.25419 20473909

[74] Loh TJ Moon H Cho S Jung DW Hong SE Kim DH SC35 promotes splicing of the C5-V6-C6 isoform of CD44 pre-mRNA. Oncol Rep (2014) 31(1):273–9. 10.3892/or.2013.2812 24173428 PMC4528307

[75] Pal S Gupta R Davuluri RV Alternative transcription and alternative splicing in cancer. Pharmacol Ther (2012) 136(3):283–94. 10.1016/j.pharmthera.2012.08.005 22909788

[76] Zhou YE O’Rourke JP Edwards JS Ness SA. Single molecule analysis of c-myb alternative splicing reveals novel classifiers for precursor B ALL. PloS One (2011) 6(8):e22880. 10.1371/journal.pone.0022880 21853052 PMC3154906

[77] Bhatla T Wang J Morrison DJ Raetz EA Burke MJ Brown P Epigenetic reprogramming reverses the relapse-specific gene expresssion signature and restores chemosensitivity in childhood B-lymphoblastic leukemia. Blood (2012) 119(22):5201–10. 10.1182/blood-2012-01-401687 22496163 PMC3369610

[78] Vicente-Duenas C Romero-Camarero I Cobaleda C Sanchez-Garcia I. Functions of oncogenes in cancer development: a changing paradigm. Blood (2013) 32:1502–13. 10.1038/emboj.2013.97 23632857 PMC3671260

[79] Chick WS Ludwig M Zhao X Kitzenberg D Williams K Johnson TE Screening for stress-resistant mutations in the mouse. Front Gent (2014) 5:310 10.3389/fgene.2014.00310 PMC4157564 25250048

[80] Carvunis AR Rolland T Wapinski I Calderwood MA Yildirim MA Simonis N Proto-genes and *de novo* gene birth. Nature (2012) 487(7407):370–4. 10.1038/nature11184 22722833 PMC3401362

[81] Aanes H Ostrup O Andersen IS Moen LF Mathavan S Collas P Differential transcript isoform usage pre- and post-zygotic genome activation in zebrafish. BMC Genomics (2013) 14:331. 10.1186/1471-2164-14-331 23676078 PMC3747860

[82] Funaki T Shunsuke K Tanabe K Natsume W Sato S Shimizu T The Arf GAP SMAP2 is necessary for organized vesivle budding from the trans-Golgi network and subsequent acrosome formation in spermatogenesis. Mol Biol Cell (2013) 24(17):2633–44. 10.1091/mbc.E13-05-0234 23864717 PMC3756916

[83] Holwerda SJB deLaat W. CTCF: the protein, the binding partners, the binding sites and their chromatin loops. Philos Trans R Soc Lond B Biol Sci (2013) 368(1620):20120369. 10.1098/rstb.2012.0369 23650640 PMC3682731

[84] Inada T Makino S. Novel roles of the multifunctional CCR_4_-NOT complex in post-transcriptional regulation. Front Genet (2014) 5:135. 10.3389/fgene.2014.00135 24904636 PMC4033010

[85] MacDonald WA. Epigenetic mechanisms of genomic imprinting: common themes in the regulation of imprinted regions in mammals, plants, and insects. Genet Res Int (2012) 2012:585024. 10.1155/2012/585024 22567394 PMC3335465

[86] Gkountela S Li Z Vincent JJ Zhang KX Chen A Pellegrini M The ontogeny of cKit+ human primordial germ cells proves to be a resource for human germ line reprogramming, imprint erasure and in vitro differentiation. Nat Cell Biol (2013) 15(1):113–22. 10.1038/ncb2638 23242216 PMC3786872

[87] Katzir Y Elhanati Y Averbukh I Braun E. Dynamics of the cell cycle network under genome-rewiring perturbations. Phys Biol (2013) 10(6):66001. 10.1088/1478-3975/10/6/066001 24162518

[88] Moore A Towards the new evolutionary synthesis: gene regulatory networks as information integrators. Bioessays (2012) 34:87 10.1002/bies.201290000 22234900

[89] Gerstein MB Kundaje A Hariharan M Landt SG Yan KK Cheng C Architecture of the human regulatory network derived from ENCODE data. Nature (2012) 489(7414):91–100. 10.1038/nature.11245 22955619 PMC4154057

[90] Stewart A Plotkin JB. The evolution of complex gene regulation by low-specificity binding sites. Proc Biol Sci R Soc (2013) 280(1768):20131313. 10.1098/rspb.2013.1313 23945682 PMC3757967

[91] Prindull G. Hypothesis: cell plasticity linking embryonal stem cells to adult stem cell reservoirs and mestatatic cancers? Exper Hematol (2005) 33:738–46. 10.1016/j.exphem.2005.03.002 15963849

[92] Skinner MK Manikham M Guerrero-Bosagna C. Epigenetic transgenerational actions of environmental factors in disease etiology. Trends Endocrinol Metab (2010) 21:214–22. 10.1016/j.tem.2009.12.007 20074974 PMC2848884

[93] Rosenberg SM Queitsch C Combating evolution to fight disease. Science (2014) 343:1088–9. 10.1126/science.1247472 24604189 PMC4117199

[94] Shapiro JA. Epigenetic control of mobile DNA as an interphase between experience and genome change. Front Genet (2014) 5:87. 10.3389/fgene.2014.00087 24795749 PMC4007016

[95] Martens JHA Mandoli A Simmer F Wierenga BJ Saeed S Singh AA ERG and FLI1 binding sites demarcate targets for aberrant epigenetic regulation by AML1-ETO in acute myeloid leukemia. Blood (2012) 120(19):4038–48. 10.1182/blood-2012-05-429050 22983443 PMC3496958

[96] Dzikiewicz-Krawczyk A Macieja A Maly E Januszkiewicz-Lewandowska D Mosor M Fichna M Polymorphisms in microRNA target sites modulate risk of lymphoblastic and myeloid leukemias and affect microRNA binding. J Hematol Oncol (2014) 7(1):43. 10.1186/1756-8722-7-43 24886876 PMC4059877

[97] Gill ME Erkek S Peters HFM. Parental epigenetic control of embryogenesis: a balance between inheritance and reprogramming? Curr Opin Cell Biol (2012) 24(3):387–96. 10.1016/j.ceb.2012.03.002 22445736

[98] Gannon JR Emery BR Jenkins TG Carrell DT. The sperm epigenome: implications for the embryo. Adv Exp Med Biol (2014) 791:53–66. 10.1007/978-1-4614-7783-9_4 23955672

[99] Feng CW Bowels J Koopman P. Control of mammalian germ cell entry into meiosis. Mol Cell Endocrinol (2014) 382(1):488–97. 10.1016/j.mce.2013.09.026 24076097

[100] Ferguson-Smith AC. Genomic imprinting: the emergence of an epigenetic paradigma. Nat Rev Genet (2011) 12:565–75. 10.1038/nrg3032 21765458

[101] Thaler F. Current trends in the development of histone deacetylase inhibitors: a review of recent patent application. Pharm Pat Analt (2012) 1(1):75–90. 10.4155/ppa.11.3 24236715

[102] Bastian L Hof J Pfau M Fichtner I Eckert C Henze G Synergistic activity of Bortezomib and HDACi in preclinical models of B-cell precursor acute lymphoblastic leukemia via modulation of p53, Pi3K/AKT, and NH-kB. Clin Cancer Res (2013) 19:1445–57. 10.1158/1078-0432.CCR-12-1511 23357978

[103] Sawicka A Seiser C. Sensing core histone phosphorylation – a matter of perfect timing. Biochim Biophys Acta (2014) 1839(8):711–8. 10.1016/j.bbagrm.2014.04.013 24747175 PMC4103482

[104] Garding A Bhattacharya N Claus R Ruppel M Tschuch C Filarsky K Epigenetic upregulation of IncRNAs at 13q14.3 in leukemia is linked to the InCis downgegulation of a gene cluster that targets NF-kBCis. PloS Genet (2013) 9:e1003373. 10.1371/journal.pgen.1003373 23593011 PMC3616974

[105] Hackett JA Sengupta R Zylic JJ Murakami K Lee C Down TA Germline DNA demethylation dynamics and imprint erasure through 5-hydroxymethylcytosine. Science (2013) 339:448–52. 10.1126/science.1229277 23223451 PMC3847602

[106] Faisal M Kim H Kim J Sexual differences of imprinted gene expression levels. Gene (2014) 533(1):434–8. 10.1016/j.gene.2013.10.006 24125951 PMC3872986

[107] Zande VDL Verhulst EC. Genomic imprinting and maternal effect genes in haploid sex determination. Sex Dev (2014) 8(1–3):74–82. 10.1159/000357146 24356125

[108] Lambertini L. Genomic imprinting: sensing the environment and driving the fetal growth. Curr Opin Pediatr (2014) 26(2):237–42. 10.1097/MOP.0000000000000072 24535495

[109] Kelsey G Feil R. New insights into establishment and maintenance of DNA methylation imprints in mammals. Philos Trans R Soc Lond B Biol Sci (2013) 368(1609):20110336. 10.1098/rstb.2011.0336 23166397 PMC3539362

[110] Tachibana M Amato P Sparman M Gutierrez NM Tippner-Hedges R Ma H Human embryonic stem cells derived by somatic cell nuclear transfer. Cell (2013) 153(6):1228–38. 10.1016/j.cell.2013.05.006 23683578 PMC3772789

[111] Hosseinpour B Bakhtiarizadeh MR Khosravi P Ebrahimie E. Predicting distinct organization of transcription factor binding sites on the promotor regions: a new genome-based approach to expand human embryonic stem cell regulatory network. Gene (2013) 531(2):212–9. 10.1016/j.gene.2013.09.011 24042128

[112] Crews D Gillette R Scarpino SV Manikkam M Savenkova MI Skinner MK Epigenetic transgenerational inheritance of altered stress responses. Proc Natl Acad Sci U S A (2012) 109:13220–4. 10.1073/pnas.1118514109 22615374 PMC3384163

[113] Liu Y Timani K Ou X Broxmeyer HE He JJ. C-Myc controlled TIP110 protein expression regulates Oct4 mRNA splicing in human embryonic stem cells. Stem Cells Dev (2013) 22(5):689–94. 10.1089/scd.2012.0271 23088399 PMC3578368

[114] Ye J Blelloch R Regulation of pluripotency by RNA binding proteins. Cell Stem Cell (2014) 15(3):271–80. 10.1016/j.stem.2014.08.010 25192462 PMC4372238

[115] Cruciat CM Niehrs C. Secreted and transmembrane wnt inhibitors and activators. Cold Spring Harb Perspect Biol (2013) 5(3):a015081. 10.1101/cshperspect.015081 23085770 PMC3578365

[116] Okumura N Akutsu H Sugawara T Miura T Takezawa Y Hosoda A β catenin functions pleiotropically in differentiation and tumorigenesis in mouse-derived stem cells. PloS One (2013) 8(5):e63265. 10.1371/journal.pone.0063265 23691006 PMC3653942

[117] Vainchenker W Constantinescu SN. JAK/STAT signaling in hematological malignancies. Oncogen (2013) 32(21):2601–13. 10.1038/onc.2012.347 22869151

[118] Griffiths DS Li J Dawson MA Trotter MWB Cheng YH Smith AM LIF independent JAK signalling to chromatin in embryonic stem cells uncovered from an adult stem cell disease. Nat Cell Biol (2011) 13(1):13–21. 10.1038/ncb2135 21151131 PMC3008749

[119] Espinoza I Miele L. Notch inhibitors for cancer treatment. Pharmacol Ther (2013) 139(2):95–110. 10.1016/j.pharmthera.2013.02.003 23458608 PMC3732476

[120] Whelan JT Hollis SE Cha DS Asch AS Adam S Lee MH. Post-translational regulation of the Ras-ERK/MAPK signaling pathway. J Cell Physiol (2012) 227(3):1235–41. 10.1002/jcp.22899 21688267

[121] Ormsbee Golden BD Wuebben EL Rizzino A. Sox2 expression is regulated by a negative feedback loop in embryonic stem cells that involves AKT signaling. PloS One (2013) 8(10):e76345. 10.1371/journal.pone.0076345 24116102 PMC3792943

[122] Fang K Han BW Chen ZH Lin KY Zeng CW Li XJ A distinct set of long non-coding RNAs in childhood MLL-arranged acute lymphoblastic leukemia: biology and epigenetic target. Hum Mol Genet (2014) 23(12):3278–88. 10.1093/hmg/ddu040 24488769

[123] Kim RJ Nam JS. Oct4 expression enhances features of cancer stem cells in a mouse model of breast cancer. Lab Anim Res (2011) 27(2):147–52. 10.5625/lar.2011.27.2.147 21826175 PMC3145994

[124] Hall VJ Hyttel P. Breaking down pluripotency in the porcine embryo reveals both a premature and a reticent stem cell state in the inner cell mass and unique expression profiles of the naïve and primed stem cell states. Stem Cells Dev (2014) 23(17):2030–45. 10.1089/scd.2013.0502 24742229

[125] Liao B Zhong X Xu H Xiao F Fang Z Gu J Itch, an E3 ligase of Oct4, is required for embryonic stem cell self-renewal and pluripotency induction. J Cell Physiol (2013) 228(7):1443–51. 10.1002/jcp.24297 23255053

[126] Tsai LL Hu FW Lee SS Yu CH Yu CC Chan YC. Oct4 mediates tumor initiating properties in oral squamous cell carcinomas through the regulation of epithelial-mesenchymal transition. PloS One (2014) 9(1):e87207. 10.1371/journal.pone.0087207 24475251 PMC3903644

[127] Zhang X Peterson KA Liu XS McMahon AP Ohba S. Gene regulation networks mediating canonical Wnt signal-directed control of pluripotency and differentiation in embryonic stem cells. Stem Cells (2013) 31(12):2667–79. 10.1002/stem.1371 23505158 PMC3830733

[128] Cheng G. Circulating miRNAs: roles in cancer diagnosis, prognosis and therapy. Adv Drug Deliv Rev (2014) 66:42–57. 10.1016/j.addr.2014.09.001 25220354

[129] Ng WL Chen G Wang M Wang H Story M Shay JW. OCT4 as a target of miR-34 stimulates p63 but inhibits p53 to promote human cell transformation. Cell Death Dis (2014) 5(1):e1024. 10.1038/cddis.2013.563 24457968 PMC4040665

[130] Peng S Maihle NJ Huang Y. Pluripotency factors Lin28 and OCT4 identify a subpopulation of stem cell-like cells in ovarian cancer. Oncogene (2010) 29:2153–9. 10.1038/onc.2009.500 20101213

[131] Jung M Peterson H Chavez L Kahlem P Lehrach H Vilo J A data integration approach to mapping OCT4 gene regulatory networks operative in embryonal stem cells and embryonal carcinoma cells. PloS One (2010) 5(5):e10709. 10.1371/journal.pone.0010709 20505756 PMC2873957

[132] Iv SR Luis E Xie X Old M Teknos TN Pan Q. Emerging role of Nanog in tumorigenesis and cancer stem cells. Int J Cancer (2014) 135(12):2741–8. 10.1002/ijc.28690 24375318 PMC4065638

[133] Meng F Forrester-Gauntlett B Henderson H Obach B 81 jak-stat signaling is critical for inner cell mass development in bovine blastocysts. Reprod Fertil Dev (2014) 27(1):133–4. 10.1071/RDv27n1Ab81

[134] Costa Y Ding J Theunissen TW Faiola F Hore TA Shliaha PV Nanog-dependent function of TET1 and TET2 in establishment of pluripotency. Nature (2013) 495(7441):370–4. 10.1038/nature11925 23395962 PMC3606645

[135] Dowell KG Simons AK Bai H Kell B Wang ZZ Yun K Novel insight into embryonic stem cell self-renewal through comparative human and mouse system biological networks. Stem Cells (2014) 32(5):1161–72. 10.1002/stem.11612 24307629 PMC4404315

[136] Wang ML Chiou SH Wu CW. Targeting cancer stem cells: emerging role of Nanog transcription factor. Onco Targets Ther (2013) 6:1207–20. 10.2147/OTT.S38114 24043946 PMC3772775

[137] Ji J Wei X Wang Y. Embryonic stem cell markers Sox2 and Oct4 expression and their correlation with Wnt signal pathway in cervical squamous cell carcinoma. Int J Clin Exp Pathol (2014) 7(5):2470–6. 24966958 PMC4069924

[138] Goissis MD Cibelli JB. Functional characterization of SXO2 in bovine preimplantation embryos. Biol Reprod (2014) 90(2):30. 10.1095/biolreprod.113.111526 24389873

[139] Yang N Hui L Wang Y Yang H Jiang X. Overexpression of SOX2 promotes migration, invasion and epithelial-to-mesenchymal transition through the Wnt/β.catenin pathway in laryngeal cancer Hep-2 cells. Tumour Biol (2014) 35(8):7965–73. 10.1007/s13277-014-2045-3 24833089

[140] Li X Xu Y Chen Y Chen S Jia X Sun T SOX2 promotes tumor metastasis by stimulating epithelial-to-mesenchymal transition via regulation of Wnt/β catenin signal network. Cancer Lett (2013) 336(2):379–89. 10.1016/j.canlet.2013.03.027 23545177

[141] Kim J Woo AJ Chu J. A Myc network accounts for similarities between embryonic stem and cancer cell transcription programs. Cell (2010) 143(2):313–24. 10.1016/j.cell.2010.09.010 20946988 PMC3018841

[142] Ghigna C Riva S Biamonti G Alternative splicing of tumor suppressors and oncogenes. Cancer Treat Res (2013) 158:95–117. 10.1007/978-3-642-31659-3_4 24222355

[143] Li X. Genomic imprinting is a parental effect established in mammalian germ cells. Curr Top Dev Biol (2013) 102:35–59. 10.1016/B978-0-12-416024-8.00002-7 23287029

[144] Elkon R Ugalde AP Agami R Alternative cleavage and polyandenylation: extent, regulation, function. Nat Rev Genet (2013) 14(7):492–506. 10.1038/nrg3482 23774734

[145] Wang X Guda C Computional analysis of transcriptional circuits in human embryonic stem cells reveals multiple and independent networks. Bio Med Res Int (2014) 2014:725780 10.1155/2014/725780 PMC3910540 24511543

[146] Yan L Yang M Guo H Yang L Wu J Li R Single cell RNA-Seq profiling of human preimplantation embryos and embryonic stem cells. Nat Struct Mol Biol (2013) 20(9):1131–9. 10.1038/nsmb.2660 23934149

[147] Marucci L Pedone E De Vicino U Sanury-Escribano B Isalan M. Cosma MP. β-catenin fluctuates in mouse ESCs and is essential for Nanog-mediated regrogramming of somatic cells to pluripotency. Cell Rep (2014) 8(6):1686–96. 10.1016/j.celrep.2014.08.011 25199832

